# Cerebro-Spinal Meningitis, or Spotted Fever

**Published:** 1865-07

**Authors:** Ephraim Ingals


### CHICAGO MEDICAL JOURNAL.

VOL XXII.] JULY, 1865. [No. 7.

### ORIGINAL COMMUNICATIONS.

### CEREBRO-SPINAL MENINGITIS, OR SPOTTED FEVER.

By EPHRAIM INGALS, M. D.

How long this disease has been known I am not able to say, but we have accounts of its prevalence in different parts of Europe, at sundry times since the last half of the 16th century. It seems to have made its unwelcome advent on this continent, in Connecticut, during the winter of 1807–8, and in a few years extended itself into several adjoining States. From that time to the present it has been recognized at longer or shorter intervals, and in many localities, but has been especially common during the last few years, occuring over a wide extent of territory, while the novelty of its nature, its severity and frequent fatality, have secu red for it the earnest attention of the profession, as is sufficiently attested by the transactions of various medical societies, and the current literature of our medical periodicals. The symptoms vary much in different cases, and yet they are numerous and characteristic enough to relieve the diagnois from any extraordinary obscurity.

A chill usually gives the first notice of the approaching disease, though this is frequently preceded by great lassitude of both body and mind. The chill is sometimes severe and protracted, sometimes light and transient, and except the case prove speedily fatal, is followed by reaction and fever. There is soreness of the throat, sometimes so slight as not to attract the notice of the patient, until attention is directed to it by the physician. Pain is the most constant and prominent symptom, usually coming early, and sometimes introducing the attack. It may extend to the whole body, or it may be limited to a part, as one or more of the joints of the extremities; or less frequently, it may be confined to the muscular tissue away from the joints. It is liable to be erratic, capriciously leaving one part only to fix itself upon another. It is sometimes light; at others it reaches a degree of indescribable anguish. It is especially likely to locate itself within the head and spine, affecting most the back part of the head and the upper part of the spine.

The sensibility of the skin, too, is often increased in a remarkable degree, and this when there is no pain except on motion or pressure. There may be delirium; and partial paralysis that results in irregular spasmodic muscular contractions is not an uncommon attendant, and this may affect the whole, or only a part of one side of the body. I have not seen paraphlegia from it. The muscular contractions are generally clonic, though they may be tonic, and these, when affecting the muscles of the back, occasion the most distressing opisthotonos but I believe this is less frequent than is generally thought. This symptom when present gives the disease a close analogy to tetanus, with which, I suspect, some cases are nearly allied. Spots appear upon the body, but less uniformly than we might be led to think from this sign having given one of the names to the disease, though I doubt not they are often present when they are not seen, for though they sometimes remain a number of days, they are frequently transient. Their color is occasionally florid, but generally dark, and sometimes the two are intermingled, and in fading away they at times pass into a greenish hue. They may be large and irregular vibices, but oftener are small, petechial, and they may be so numerous as to give to the surface a mottled appearance. They come early in the disease; they are sometimes elevated, but usually not; they do not disappear under pressure, and cut down upon are found to involve the deep layers of the skin, and appear to be occasioned by the escape of blood from the vessels; and in two fatal cases I have seen the hemorragic tendency so well pronounced, that blood exuded from the mucous surfaces of the mouth, nasal passages and vagina, caused apparently by a dissolution of this fluid. In some cases vomiting is an early and distressing symptom, seemingly not due to any gastric derangement, but emenating from a morbid impression made upon the nervous system, and if this is protracted and obstinate, I look upon it as of inauspicious significance. The function of the kidneys is apt to be deranged, and for a time there may be less than the normal amount of secretion, and this may be followed by a natural or even copious flow of urine, of a dark color, which, when left to stand until it is cold, frequently gives an abundant, flocculent, pinkish sediment, which disappears again when heat is applied, and which I suppose to be the urate of ammonia. The urine, too, is sometimes tinged with blood. The pulse is soft, perhaps full, but easily compressible, and shatters under the finger as if the artery contained a fluid less cohesive than blood; it is frequently intermittent; it may be rapid or very slow, and I have seen these changes so sudden that there would be a variation of twelve beats in the different quarters of the same minute. The bowels are generally constipated, but sometimes the reverse; and the appetite and powers of digestion occasionally remain in a remarkable degree, and I have never seen a case where the bowels themselves were the seat of pain. Its symptoms are said sometimes—especially with children—to simulate cholera morbus, but I have seen no such cases. The tongue is moist, and generally, but not always, lightly covered with a whitish coat. The disease is attended with but little fever, nor is there much thirst.

Delirium, followed by coma and profuse cold sweats, are symptoms that awaken the gravest apprehensions. The special senses of sight and hearing are sometimes lost. The diseases for which it is most likely to be mistaken are rheumatism and inflammation of the brain.

Among the most interesting considerations relating to this affection, is the question as to its essential nature—that is, is it inflammatory, or is it not? does it belong to the sthenic or the asthenic type? My own belief is that it first effects the nervous system in such a way as to impair its energy, and that in most cases, the blood speedily becoming involved tends to dissolutiou; that it belongs to the asthenic type of disease; and that while inflammation of the meningies of the nervous centres sometimes occurs, it is only an infrequent and accidental complication, and is by no means the essence of the malady. I have made no post-mortem examinations, and am therefore obliged to rely for the pathology upon the reports of others, and from conclusions drawn from observing its tendency and progress, and especially of the influence upon it of therapeutic agents.

Few autopsies appear to have been made of those dying of this disease; but fortunately some of these have been conducted with great intelligence and recorded with praiseworthy fidelity. The little work on Spotted Fever, by Elish North, published in 1811, and which contains a very valuable history of the epidemic which prevailed just before that time in New England, gives the result of the examination of five cases. In three of these there was no inflammation, and in the other two it was slight, and entirely subordinate to pathological conditions of an opposite type.

In the annual volumes of the *American Medical and Philosophical Register*, published from 1810 to 1814, considerable space is given to observations on this disease; or at least to one very nearly akin to it, but in which the weight of the malady fell upon the lungs, more than upon the brain; but no valuable post-mortem examinations are recorded.

Dr. Jewell, in the *American Journal of the Medical Sciences*, for July, 1864, and Dr. Lidell, in the same journal for January, 1865, have given us the most interesting and instructive reports that I have seen. Without too much extending this article by particularizing from these or other sources, I may briefly say that the pathological post-mortem conditions have generally been found as follows. The vessels of the scalp and of the meningies of the nervous centers are abnormally full, while the blood in all parts of the body is dark, fluid and uncoagulable. The substance of the brain is nearly natural; while its ventricles, the sub-arachnoid space, spinal canal, and pericardium generally contain much serum, either clear or sanious. The kidneys are congested; and the same may be said of the lungs, while in their substance blood has been extravasated, and purpuric spots have been noticed on the mesentery.

Any opinion as to its causes would partake much of what is speculative and conjectural. It seems to be of zymotic origin—it prevails most in damp, cold weather, but is not confined to any season, temperature, or known atmospheric conditions. It follows the general law of epidemics, in that it is most fatal on its first appearance, for the reason, I suppose, that the systems most predisposed to receive the poison, and which as a consequence are first attacked, best supply the conditions to develop its virulence. Having the disease once does not protect the system, for I have treated one patient for it in two separate attacks. That it is contagious—but in a less degree than a number of diseases—I have no doubt. In some cases in which its contagious origin was most clearly indicated, the period of incubation was from ten to fourteen days. Cases occurring in from two to five days after exposure, have always been surrounded by circumstances to make me suspect that the disease was developed from epidemic influences. It has been designated by different names; generally in Europe as thphoid meningitis, but in this country, mostly, by the appellations at the beginning of this article; but with these, it is clear enough from what has been written and said, the profession are little satisfied. Its etiology and pathology are perhaps not well enough understood to enable us to designate it in conformity with its essence and nature, and under such circumstances we should be most fortunate in the adoption of a purely arbitrary name, that should not pretend to indicate the character of the disease. Of the two names under which I have spoken of it, that of Spotted Fever is best—for nothing can be more palpable than that this is in no way descriptive of the disease, for in a large percentage of cases no eruption is observed, and I know of no serious malady that lights up so little fever in the economy, and therefore this has much of the merit of an arbitrary name. It is less easy to justify the use of the term cerebro-spinal meningitis, or paliate the objections against it, for this name seems equally inappropriate, while it embodies the misfortune that it is liable sadly to mislead us in the application of remedies. It fixes the seat of the disease in the cranium and spinal canal, and thus while the patient lives, beyond the scrutiny of our senses, while the last syllable—*itis*—indicates its nature in the most unequivocal manner, and in a way likely to exert a a baneful influence on the adoption of a plan of treatment by one not familiar with it, there is no greater reason to conclude that the disease is of an inflammatory type, because of the tendency to serous or sanious effusion, than that the pathological condition to which cholera is due is inflammation of the bowels.

The prognosis will depend much upon the character of the prevailing epidemic, for some are much more fatal than others; but it should always be cautious, for the symptoms occasionally assume a sudden and unexpected virulence—but while it is cautious, it should at the same time be hopeful, for sometimes the patient steps back from the very embrace of the grave.

The question of treatment—the most practical and important of all—remains to be considered. I rely in this, as in most other diseases, on a small number of remedies; but I think success depends very much on the manner in which these are used, but perhaps as much on the treatment we omit as on that we apply. The chill requires to be treated, as all initial chills should be, by heat, internally and externally, and the use of gentle stimulants to bring about speedy reaction. When pain is severe, it should be subdued, and for this purpose no remedy approaches opium in power and usefulness. I am disposed to think that upon these cases opium has some specific influence beyond its power of controlling pain. Its superior value above every other remedy is attested by the best observers in all epidemics of the disease, both in this country and in Europe. If, however, with severe pain in the head, there should be great heat, the countenance suffused, the pulse not only full, but strong, then we might fear an inflammatory tendency and withhold the opium, or use it with caution. Ordinarily it should be pushed until the desired effect is obtained, and this will generally require large and repeated doses, partly in consequence of the severe pain neutralizing to some extent the natural action of the remedy, and partly perhaps because it may be curative as an antidote, and therefore is required in quantity proportionate to the morbific influence to which the particular case is due. Sulphate of Morphia, or its equivalent of any other opiate, may be given in the dose of one-fourth or one-half a grain, at intervals of from one to six hours, according to the degree of pain and the effect of the remedy. Rubefacients and epispastics are most important adjuvants in overcoming the pain, while they also exert a beneficial influence as stimulants. They do most good applied upon the spine and nape of the neck. For the local muscular pains, Prof. Allen recommends the hypodermic opiate injection, from which I should expect an excellent effect, but have never verified this by experience. Upon the first indications of a decline of the vital powers, as shown by the flagging of the circulation, the pulse being weak, compressible, rapid or intermittent; a tendency to profuse perspiration; a blunting of the mental faculties, or loss of the special senses; then alcoholic stimulants should be pressed with the same vigor and diligence as the opium has been, and as in the use of this article, these should be given until their effects are shown in the abatement of the symptoms just enumerated, when the pulse will be firmer and more natural, the heat of the surface greater, the perspiration less, while the faculties of the mind will be aroused.

As to alcoholic stimulants, the practitioner should exercise the most sleepless vigilance. It is better to use them in advance, than to wait the establishment of the evidences of depression. They should always be administered as a precautionary measure, whenever they may be with safety. They should be given if they do no harm; and herein I would violate what I think should be a general rule, namely: to withhold medicines that are not clearly demanded; believing in general that in this particular, omission is less likely to be an error than commission. Wines are the best stimulants; and of these, Sherry, Madeira and Champaigne are to be preferred; while the distilled liquors, brandy and whisky, are excellent when they agree with the stomach, and are readily absorbed. I have said that the quantity to be administered must depend upon the effect produced, and therefore, until the desired effects are obtained, I would have the stomach constantly supplied to the limit of its capacity of absorption. Some suppose they are stimulating such cases when they administer a teaspoonful or tablespoonful of wine or brandy every hour. Many times this is little better than to do nothing. Good care too must be taken not to discontinue the remedy too soon, for the patient must be constantly watched and carefully supported until the vital powers are re-established.

To accomplish these results, large quantities of stimulants may be required. Dr. Wilson, as quoted in North's work, found a patient to improve on the use of one pint of wine in an hour, to decline when it was withheld, and rapidly to improve when it was again freely administered. Dr. Smith "sometimes allowed his patients two bottles of Madeira in the day; and in one instance the patient took two bottles of port in little more than half that time." Dr. Bestor gave "two quarts of brandy and one quart of wine in twenty-four hours, besides twenty drops of tinct. of opium once in two hours." Drs. Haskell, Spooner and Holmes "gave to a girl twenty years old one quart of brandy and twenty grains of good Turkey opium in twelve hours, besides external stimulants." Dr. Strong "gave one quart of brandy in eight hours to a delicate female, and a grain and a half opium every two hours, and a very liberal use of brandy and opium was found necessary for a number of days, and should we mention the quantity which she actually took, our account would hardly gain credit." Dr. Lyman, in seven hours, "gave one pint and a half of wine, two ounces of brandy and other stimulants."

I have quoted thus lengthily to show to what extent these remedies may, under certain circumstances, be carried with advantage; and it is noticed that they do not occasion intoxication. Such teachings, of course, would apply only to extreme cases, and not to many milder ones which tend to speedy recovery and demand but little treatment. If the extremities are cold, they should be made warm, and for this purpose dry heat is easiest of application and best for the patient. Bottles filled with hot water and enveloped in cloths are the most convenient means. I dislike much sweating, on account of its cooling tendency, while it increases the weakness of which it is a symptom. It is better that the surface should be at a pretty high temperature, and dry. If there is great heat of the head, apply cold, but beware of too much reducing its temperature, for this favors effusion, which is a circumstance the most to be dreaded.

For the vomiting, opium, ether and camphor are the best remedies. Associated with the means above enumerated, I have administered the bicarbonate of potash in the dose of ten or fifteen grains, every four or six hours, until from four to eight drams are taken, and I think with advantage. It at least has the merit of being harmless—notwithstanding the theoretical notions of some about the action of such remedies upon the blood.

The bitter tonics are useful, but have no specific control of the disease; they only lend some support. Cinchona, gentian and calumba are the best. I am afraid to use the sulph. of quinine, except as a tonic, in the dose of ½ to 1 gr., and then it is not so good as the fluid extract, or tinct. of cinchona, or other of the bitter tonics. I know that recoveries have followed its use in large doses; but so far as I have seen, the cerebral symptoms are aggravated by its use in this manner. I have said it is quite as important to know what to avoid as what to do. Above all things use no depletion. I perhaps do not need to caution against drawing blood; for it is not now the fashion to bleed even in inflammatory and plethoric diseases. I do not think we bleed enough, for the pendulum of custom and habit, in its vibration through its arc, has reached the extremity of absolute prohibition. If this disease was what some suppose it to be, then bleeding would be judicious practice; but it is not, and nothing is more destructive than to loose blood. This was soon found out in the epidemics fifty years ago, when bleeding being in vogue, many tried it, and found their patients to sink with appalling certainty and rapidity. Among all who have written, I have seen hardly a conflict of opinion about this. Blood then should neither be drawn by the lancet nor leeches.

This might well teach us caution in the use of depletion in the mode that the present has more generally substituted for the lancet of the past—that is by cathartics. If the disease is not inflammatory; if it is not of the sthenic type; if it does not require depletion, then I know not upon what principle we should use cathartics, except as eliminatives; and observation, so far from confirming their utility, would teach us caution in their administration. If the secretions into the bowels are offensive, then they should be removed by the most gentle laxatives; but if they are not and the bowels seem healthy, leave them alone. If the appetite remains, as it sometimes does, give good food; and in any event, nourish the system as well as the organs of digestion will permit, and animal food is usually best.

No disease requires such vigilant watchfulness; for a few hours will precipitate from the brightest hope to trembling anxiety or the deepest despair. Some supposing it to be due to a blood poison, are moved by theoretical considerations to rely upon chemical antidotes to the *materies morbi;* but theories do so much to blind the perception and mislead the judgment that they should be followed with distrust, unless unbiased observations of the effects of remedies confirm the truth of their teachings.

